# Anomalies in neurovascular coupling during early diabetes: A review

**DOI:** 10.1111/ceo.14190

**Published:** 2022-11-18

**Authors:** Erica L. Fletcher, Michael A. Dixon, Samuel A. Mills, Andrew I. Jobling

**Affiliations:** ^1^ Department of Anatomy and Physiology The University of Melbourne Melbourne Victoria Australia; ^2^ Department of Anatomy and Developmental Biology Monash University Melbourne Victoria Australia

**Keywords:** angiotensinogen, diabetic retinopathy, microglia, Müller cell, neurovascular coupling

## Abstract

Diabetic retinopathy is the most feared complication for those with diabetes. Although visible vascular pathology traditionally defines the management of this condition, it is now recognised that a range of cellular changes occur in the retina from an early stage of diabetes. One of the most significant functional changes that occurs in those with diabetes is a loss of vasoregulation in response to changes in neural activity. There are several retinal cell types that are critical for mediating so‐called neurovascular coupling, including Müller cells, microglia and pericytes. Although there is a great deal of evidence that suggests that Müller cells are integral to regulating the vasculature, they only modulate part of the vascular tree, highlighting the complexity of vasoregulation within the retina. Recent studies suggest that retinal immune cells, microglia, play an important role in mediating vasoconstriction. Importantly, retinal microglia contact both the vasculature and neural synapses and induce vasoconstriction in response to neurally expressed chemokines such as fractalkine. This microglial‐dependent regulation occurs via the vasomediator angiotensinogen. Diabetes alters the way microglia regulate the retinal vasculature, by increasing angiotensinogen expression, causing capillary vasoconstriction and contributing to a loss of vascular reactivity to physiological signals. This article summarises recent studies showing changes in vascular regulation during diabetes, the potential mechanisms by which this occurs and the significance of these early changes to the progression of diabetic retinopathy.

## 1 INTRODUCTION

Diabetic retinopathy remains one of the most feared complications for those with diabetes. It develops in ~22% of those with type I and II diabetes, or an estimated 103 million people world‐wide.^1^ Vision‐threatening forms of diabetic retinopathy, including diabetic macular oedema or proliferative diabetic retinopathy develop in ~10% of those with diabetes. Although there have been tremendous efforts to improve clinical care and treatment of those with diabetic retinopathy, over the last 30 years the burden of diabetic retinopathy has continued to increase, particularly in developing nations.^2^ Reflecting this, an increase in prevalence of diabetic retinopathy of 14.9% was identified in southeast Asia, and sub‐Saharan Africa, whereas over the same period of time and in the same countries there was an overall decrease in prevalence of vision impairment due to glaucoma or age‐related macular degeneration.^2^ Understanding the underlying mechanisms that lead to the development and/or progression of this disease is important for developing novel therapies to improve clinical management.

The visible vascular pathology that develops during diabetic retinopathy is well described and central to diabetic retinopathy management.^3^, ^4^ Formation of new blood vessels via angiogenesis, and/or vascular leakage leading to macular oedema are well described causes of irreversible vision loss in those with diabetic retinopathy.^4^ However, there are also a range of other more subtle changes in vascular and neural function that occur from a very early stage of diabetes.^3^, ^5^ Retinal function, as measured by multifocal electroretinography, is known to be reduced in areas that later develop visible vascular pathology, suggesting that neural dysfunction precedes the development of visible vascular pathology.^6^, ^7^ In addition to multifocal electroretinogram alterations, other studies have also reported early deficits in contrast sensitivity and dark adaptation.^5^, ^8^ More recently, a meta‐analysis of 36 studies examining the thickness of retinal layers with optical coherence tomography showed significant thinning of the macula ganglion cell layer and nerve fibre layer in those with diabetes who were yet to exhibit clinical signs of retinopathy.^9^ Moreover, gradual loss of the inner retina (ganglion cell layer‐inner plexiform layer) has been shown to independently predict long‐term progression of diabetic retinopathy in those with type II diabetes.^10^ In addition to inner retinal change, a recent study showed patients with no signs of diabetic retinopathy exhibited photoreceptor change as measured by optical coherence tomography.^11^ These human studies have generally been recapitulated in animal models of diabetes with both inner and outer retinal neuronal change reported. While these studies highlight the myriad of early changes that occur in the diabetic retina, the aetiology of these alterations remains to be determined.

The metabolic needs of neurons in the retina and brain are precisely met by functional changes in the vasculature to maintain normal neural function. This localised control is referred to as neurovascular coupling – a process whereby blood flow is altered in direct response to neural activity. Approximately 20% of total body energy consumption is attributed to neural activity, and synaptic activity, in particular, is highly energy demanding. Thus, when even slight changes to the supply of energy metabolites to neurons occurs, dysfunction and loss of neurons can be a consequence. In the brain, aberrant neurovascular coupling and reduced cerebral blood flow have been implicated in Alzheimer's disease, multiple sclerosis, traumatic brain injury, spinal cord injury and stroke.^12^, ^13^ In the retina, disruption of neurovascular coupling has been implicated as a possible contributing factor to the development of diabetic retinopathy.

Unlike other parts of the body, where vascular supply is regulated by the autonomic nervous system, in the Central Nervous System and retina, blood flow is precisely regulated by non‐neural means. In particular, neurovascular coupling is mediated via the neurovascular unit, a community of diverse cells types including neurons, glia, microglia and endothelial cells. While the contribution of glia in modulating vascular calibre has been demonstrated in both the brain and retina, these cells only regulate parts of the retinal vascular tree. Indeed, there are likely to be multiple cellular mechanisms that regulate blood flow in response to neural needs.

The aim of this review is to provide a detailed overview of the cellular mechanisms important for regulation of retinal vascular function, and in particular, to highlight the contribution that microglia have maintaining proper retinal blood flow in both health and diabetic retinopathy.

## 2 RETINAL VASCULAR CHANGES THAT DEVELOP FROM AN EARLY STAGE OF DIABETES

The metabolic needs of the neurons of the retina are met by the actions of a dual vascular supply, including vessels located within the choroid (choroidal vasculature) and the inner retinal vasculature derived from the central retinal artery. The choriocapillaris provides the majority of nutrient support for photoreceptors and does not autoregulate, suggesting that regardless of retinal activity, nutrient flow to photoreceptors remains fixed. In contrast, inner retinal neurons (proximal from the outer plexiform layer) are supplied by the arterioles and capillaries that arise from the central retinal artery and fan out to form three parallel plexuses within the ganglion cell layer (called the superficial vascular plexus), the border of the inner nuclear and inner plexiform layers (called the intermediate vascular plexus) and the border of the inner nuclear and outer plexiform layers (called the deep vascular plexus).^14^ As this vascular supply is critical to homeostatic retinal function and diabetes is often described as a vascular disease, it is not surprising that there is considerable functional and structural evidence that retinal vasculature is altered from a very early stage of diabetes.

One of the earliest changes in vascular function that occurs in those with diabetes is reduced blood flow, which is reported within 5–10 years following the onset of diabetes.^15^, ^16^ Reductions in blood flow have been reported prior to the development of any other signs of disease in both humans and also rodent models of diabetes, implying that it may be a potential driver of subsequent retinal pathology.^17^, ^18^ In agreement with the observations that blood flow is altered in diabetes, vascular calibre has also been shown to be altered in those with diabetes.^19^ These measures have focused on changes in the largest arterioles and venules within one disc diameter of the optic nerve head, quantified using computerised analysis of fundus images. Notably, evaluation of subjects in the Wisconsin Epidemiology Study of Diabetic Retinopathy revealed that larger diameter veins were associated with increased risk of progression of diabetic retinopathy.^19^, ^20^, ^21^ In contrast, a study of Australian children with type I diabetes showed that larger arteriolar diameters were associated with increased risk of diabetic retinopathy over a 2.5 year follow up period.^22^ More recently, optical coherence tomography angiography (OCTA) has been utilised to evaluate not just the largest blood vessels near the optic nerve head, but also capillaries in the deeper layers of the retina. These studies show that there is a reduced density of capillaries within the superficial and deep plexuses in those with proliferative diabetic retinopathy compared with no retinopathy^23^, ^24^ and in one study of 34 subjects with type I diabetes, density loss of capillaries preceded loss of neuronal layers, suggesting that vascular compromise could be one of the earliest identifiable changes in the retina of those with diabetes.^25^

In the early pre‐clinical stage of diabetic retinopathy, the coincident reduction of retinal blood flow, neuronal dysfunction in the inner retina, and glial/microglial cell reactivity suggests a breakdown in the neurovascular unit early in diabetes,^18^, ^26^ Several studies in humans and animals have demonstrated a diminished or absent light flicker response in blood vessels, beginning before the appearance of any signs of retinopathy, and increasing with DR severity.^18^, ^26^, ^27^ The neuronal effects induced by the flicker light response can also be detected using the ERG. One study by Lasta et al. (2013) used the dual response of neuronal and vascular response to flickering light to test the neurovascular response in blood vessels in conjunction with neuronal ERG responses in type 1 diabetes patients without diabetic retinopathy.^28^ Lasta et al. found that the blood vessel response to flicker stimulus was reduced early in patients, however, this was seen independently of any ERG deficits,^28^ indicating that abnormal neurovascular coupling presents prior to neuronal functional decline.

**FIGURE 1:**
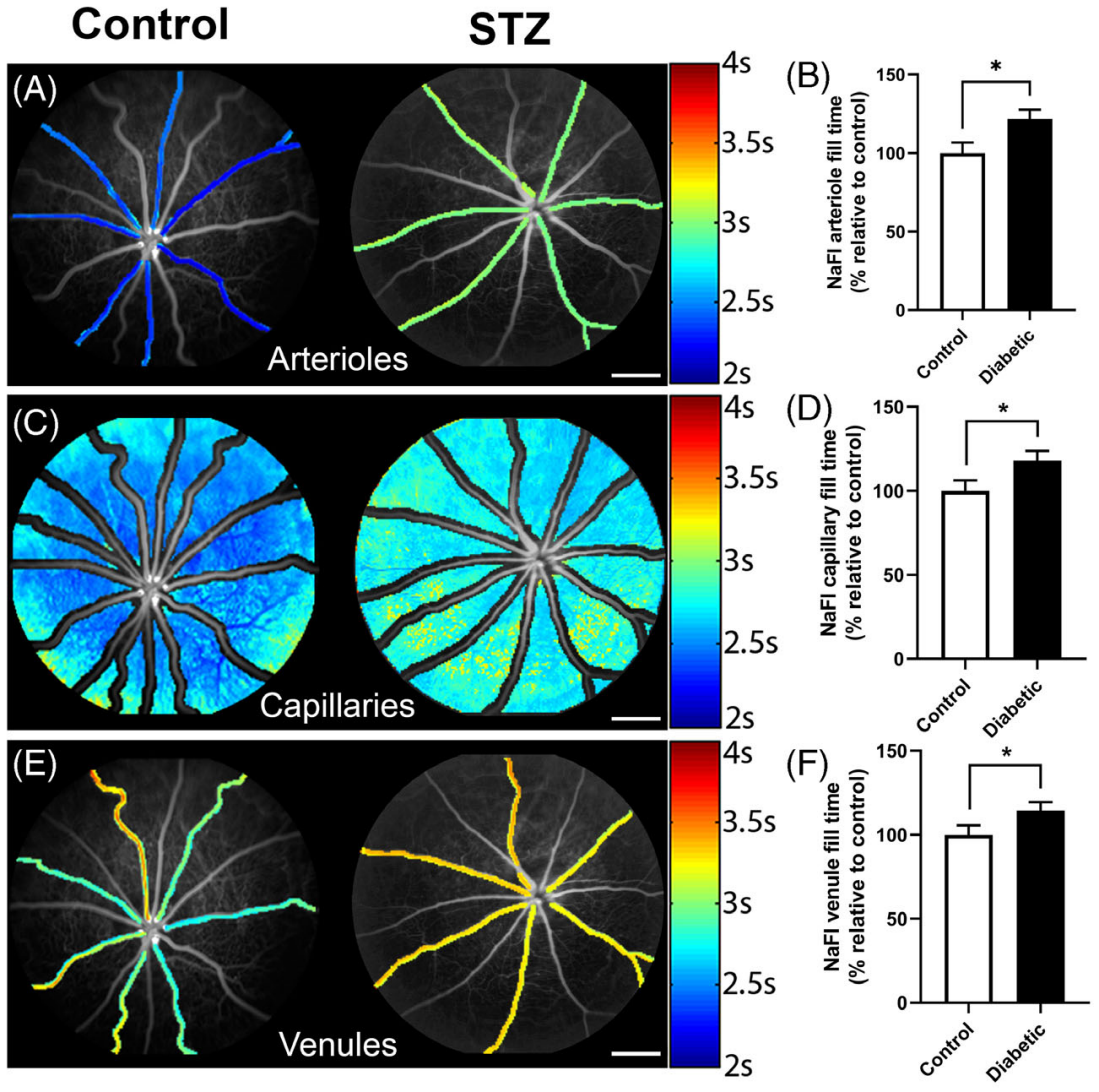
Retinal blood flow is reduced in arterioles, capillaries and venules in the retina during diabetes. Fluorescein angiograms of control and STZ induced diabetic rats showing the blood flow in arterioles, (A) capillaries (C) and venules (E). Rate of flow is indicated by the colour map to the right. Graphs of the mean + SEM fill time in (B) arterioles, (D) capillaries and (F) venules for control and diabetic rats 12 weeks following onset of diabetes. Fill time is expressed as a percentage of the control and shows that it is significantly longer in the diabetic retinae compared to control. *N* = 10 per group; *p < 0.05 unpaired *t* test. *Source*: Figures are adapted from Reference^29^

Our recent data of vascular regulation in a rodent model of type I diabetes showed that retinal blood flow was reduced from 4 weeks following the onset of diabetes.^29^ Figure 1 shows blood flow changes in the rat retina as measured by video fluorescein angiography across arterioles, capillaries and venules. Following 4 weeks of diabetes (induced with streptozotocin), blood flow is reduced (or the fill time increased) across all three vessels types.^29^ This was demonstrated using two methods to measure blood flow including video fluorescein angiography as well as high‐speed imaging to measure movement of blood cells within large arterioles and venules within the superficial vascular plexus. Importantly, both methods showed that blood flow was reduced in both arterioles and venules independent of a change in the diameter of these larger vessels. By using optical coherence tomography angiography, we found that at this early time post diabetes induction there was a significant reduction in the diameter of the small capillary vessels within the superficial plexus.^29^ This was independent of any loss of capillary density suggesting early loss of capillary vasoregulation via impaired neurovascular coupling could precede the capillary loss and contribute to the reduced blood flow found in diabetic retinae. These are important findings that suggest it is possible to study the cellular basis for the reduction in retinal blood flow using animal models and furthermore, such studies may be of significance for the progression of disease. Moreover, the techniques described above are similar to those used in clinical management of patients, suggesting that the knowledge developed in animal models may directly inform the assessment of disease progression in a clinical setting.

## 3 THE CONTRIBUTION OF PERICYTES TO NEUROVASCULAR COUPLING AND THEIR POTENTIAL ROLE IN EARLY VASCULAR DYSFUNCTION DURING DIABETES

**FIGURE 2:**
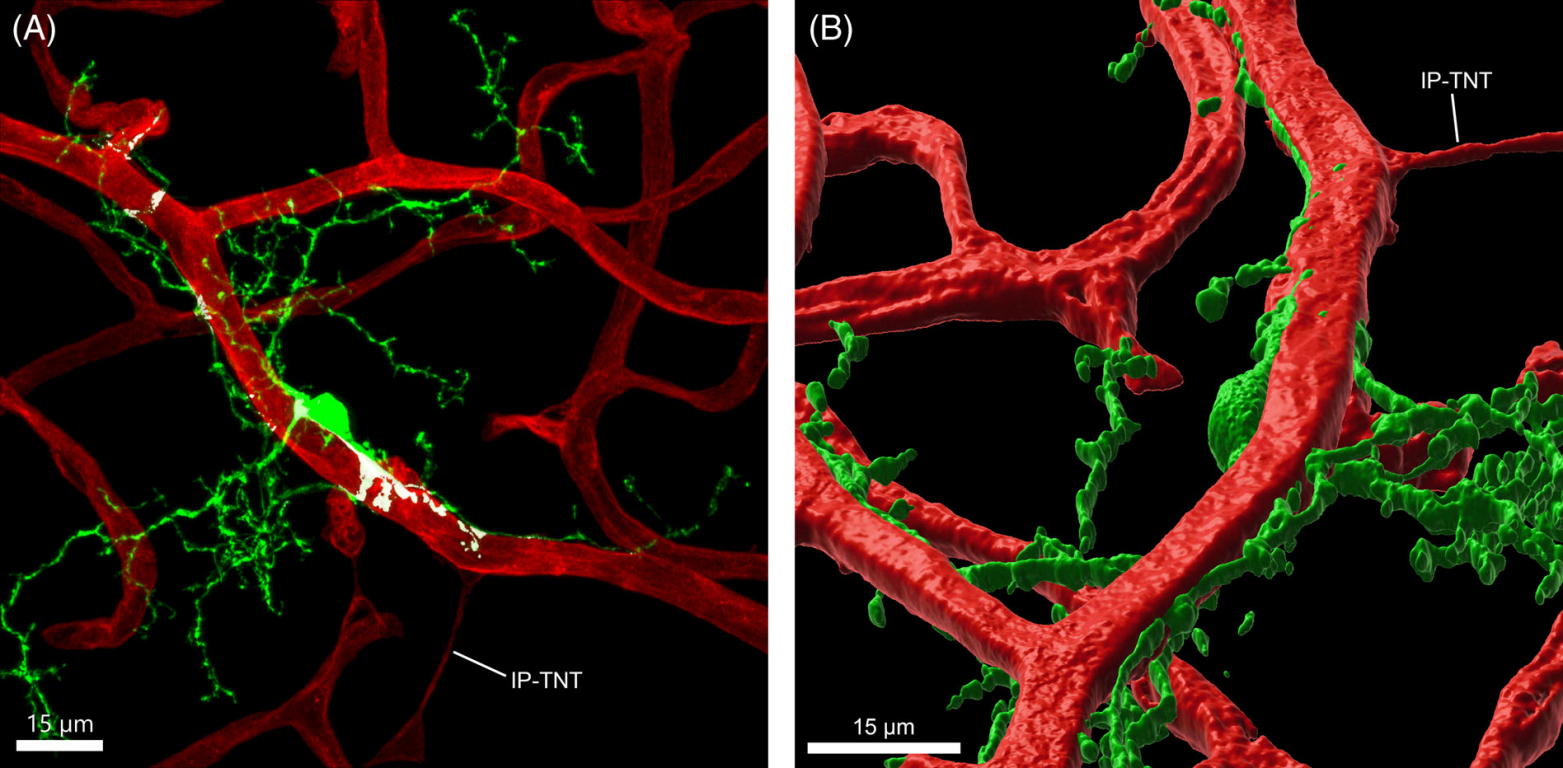
Microglia are closely associated with retinal vasculature. (A) Whole‐mounted retina from the Cx3cr1+/GFP mouse retina imaged at the level of the superficial and intermediate vascular plexuses labelled for blood vessels (IB4, red) and microglia (GFP, green). Contact between the microglial cell and vasculature is highlighted in white, determined by the apparent co‐localisation of fluorescence. An interpericyte tunnelling nanotube (IP‐TNT) can be seen connecting capillaries of the superficial vasculature with microglial contact at the distal end. (B) A 3D reconstruction of the image in (A). Note this cell has been inverted so that the areas of contract between the microglia and the blood vessel are apparent.

Pericytes are contractile cells that lie on the abluminal side of capillaries and show several different morphological subtypes often dependent on where on the capillary they are found.^30^, ^31^ While the capacity of these cells to control vessel calibre and blood flow has been controversial, it is now accepted that pericytes can actively constrict and dilate capillaries independent of arterioles via a calcium‐dependent process.^32^ Within the retina, studies have shown vasoactive factors such as Angiotensin II, Endothelin and ATP can modulate pericyte mediated vessel diameter,^33^ with signalling through the prostaglandin E2 receptor 4 (EP4) important for pericyte mediated vasodilation.^32^ While the contractile machinery responsible for pericyte mediated capillary regulation is still being elucidated, recent work has suggested that apart from the localised capillary response, pericytes also are capable of a more coordinated response. Work from Alarcon‐Martinez and colleagues has shown that within the retina, diversion of blood from one part of the vascular tree to another involves interpericytes communication via extremely fine processes called interpericyte tunnelling nanotubes (IP‐TNTs).^34^ Specifically, they show that when one pericyte contracts, the neighbouring blood vessel dilates due to communication through IP‐TNTs. Importantly, when IP‐TNTs are selectively ablated or affected by disease (e.g., retinal ischaemia, glaucoma), vascular regulation is impaired.^34^, ^35^ An example of an IP‐TNT is shown in Figure 2, as a fine structure connecting two capillaries.

Being involved in capillary regulation and blood retina barrier maintenance, it is perhaps not surprising that pericytes are regarded as one of the first vascular cell types affected by diabetes. Anomalies in pericyte density and function are known to occur in humans with diabetes and considered an important step in the initiation of early diabetic retinopathy. Pericyte loss and the appearance of ‘pericyte ghosts’ is considered to be one of the earliest signs of diabetic retinopathy^36^ with human post‐mortem studies identifying pericyte loss as a significant predictor of microaneurysms.^37^ Providing some mechanistic basis for this cellular loss, some patients with diabetes develop autoantibodies targeting pericytes. For example, a recent study identified an increase in pericyte reactive IgGs in 44 individuals with type II diabetes and diabetic retinopathy. Notably, the pericyte reactive IgGs activated complement especially C5a in those with diabetic retinopathy implicating innate immune mechanisms in the loss of pericytes.^38^ Apart from cell loss, studies have also showed altered pericyte function during diabetes with pericytes obtained from diabetic and ‘normal’ donor tissue showing a reduced contractile phenotype and altered cytoskeletal signalling (F‐actin and smooth muscle actin).^39^ Rodent models of diabetes show similar pericyte effects with a 70% reduction in pericyte‐endothelial cell connectivity^40^ and a 15% reduction in density within 6 months of onset of diabetes.^41^ Furthermore, functional deficits in rodent pericytes have been reported by 8–12 weeks following the onset of diabetes, with interpericyte communication reduced because of a loss of gap junctions, especially those expressing Connexin 43.^42^

In summary, pericytes are known to be critical for regulating vascular calibre and are a key cell type that are altered during early stages of diabetes. Obviously, any loss of pericytes on the retinal capillary network or altered contractile capacity would impact upon local vessel control. For this reason, the cells and mechanisms that lead to functional change of pericytes is an important consideration for understanding early vascular changes in diabetes.

## 4 THE ROLE OF MACROGLIA IN REGULATING VASCULAR FUNCTION DURING DIABETES

As mentioned previously, neurovascular coupling and subsequent vessel regulation involves signalling between neural synapses and the vasculature via the neurovascular unit. While pericytes are directly located on the vessel, proper coordination needs the involvement of support cells (glia) that span from neuron to vessel. In the brain, processes of astrocytes are known to contact both blood vessels, as well as neural synapses. This was recently confirmed in vivo, in a study of brain astrocytes in transgenic mice.^43^ Using high resolution two‐photon imaging through a cranial window, the vascular contacts astrocytes was assessed, revealing virtually all astrocytes have contact with at least one blood vessel, and a majority contact at least three vessels.^43^ Moreover, changes in brain astrocyte function (as detected by changes in intracellular calcium) are associated with vasodilation or vasoconstriction of neighbouring blood vessels.^44^ The mechanisms by which neurons signal their metabolic needs to the vasculature is thought to involve neurotransmitter signalling to glia. Indeed, astrocytes are known to express receptors for a range of neurotransmitters, including glutamate, GABA, norepinephrine, acetylcholine, ATP, and endocannabinoids, which can induce a change in intracellular calcium within astrocytes.^45^ In response, astrocytes have the ability to signal directly to vasculature via release of vasoactive factors, such as arachidonic acid (AA) metabolites, prostaglandin (PG) E2 and epoxyeicosatrienoic acids (EETs), and 20‐HETE.^46^

Inner retinal vessels, like blood vessels of the brain, are devoid of input from the autonomic nervous system and similar to the brain, the retina also contains several support cells, including astrocytes, Müller cells and microglia that form connections with inner retinal blood vessels. However, unlike the brain, retinal astrocytes do not have processes that extend into the synaptic regions of the retina and are therefore unlikely to have a role in retinal vascular modulation. Rather, in the retina both Müller cells and microglia are known to contact *both* blood vessels and neural synapses and therefore have a potential role in regulating vascular function in response to neural activity.^29^

**FIGURE 3:**
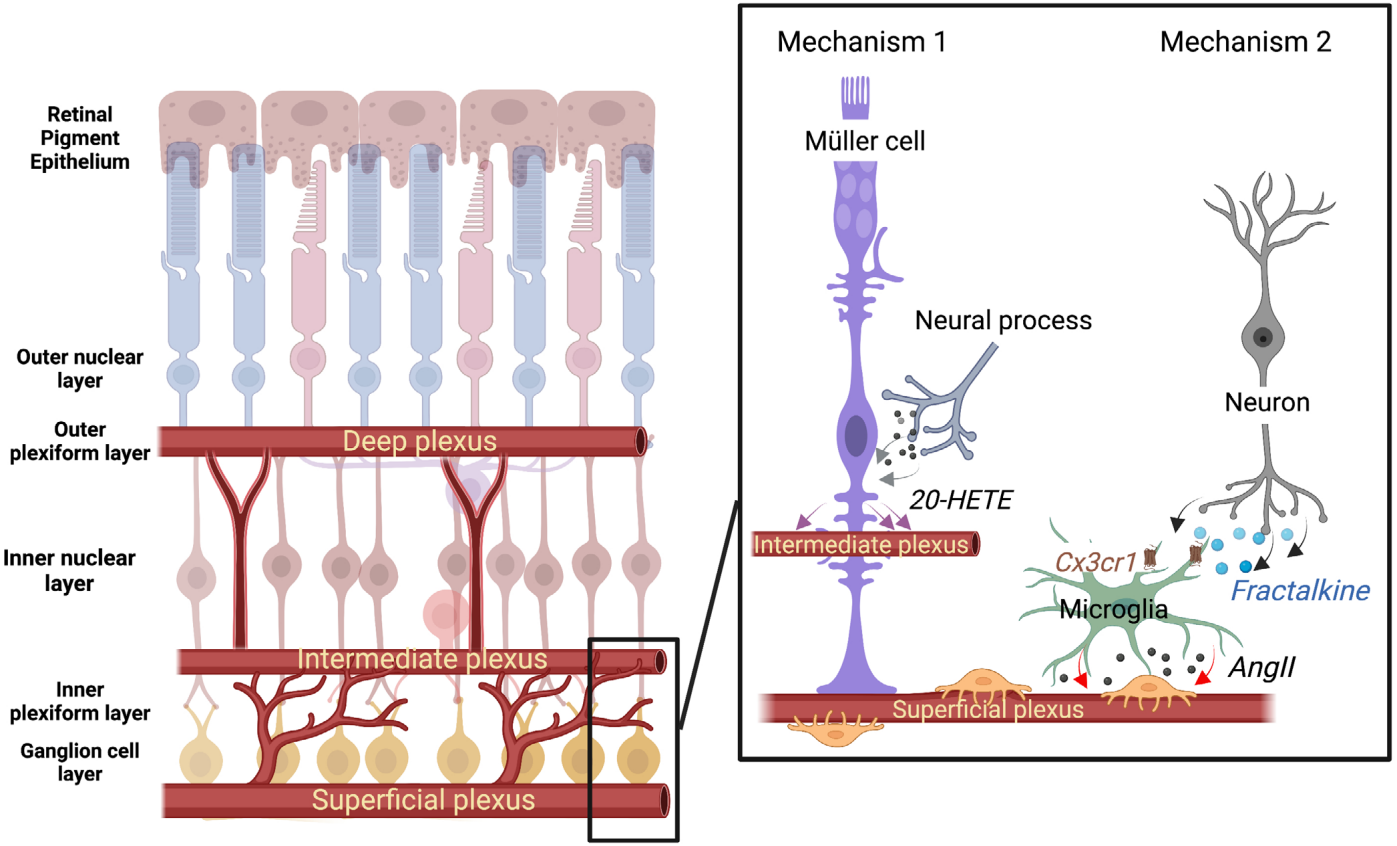
Schematic of Müller cell and microglia control of the inner retinal vasculature. A schematic diagram is shown highlighting the three major plexus of the inner retinal vasculature (deep, intermediate and superficial plexus). Vascular control of the superficial and intermediate plexus involves at least two mechanisms, shown to the right. First, Muller cells regulate the calibre of the vessels in the intermediate plexus in response to neural needs via eicosanoids, such as 20‐HETE. In contrast, vessels of the superficial plexus have extensive contact with microglia. They induce vasoconstriction in response to fractalkine‐mediated release of Angiotensin II.

Light stimuli are known to induce vasodilation or vasoconstriction of neighbouring arterioles especially in the intermediate plexus.^47^, ^48^ There have been a number of studies examining whether Müller cells are capable of regulating the retinal vasculature in response to neural function.^48^, ^49^ These studies show that the mediators of vasoregulatory function in the retina are similar to those in the brain and involve arachidonic metabolites such as EETs, and 20‐Hydroxy‐5, 8, 11, 14‐eicosatetraenoic acid (20‐HETE).^48^ More recently functional connection between glia and the vasculature has been extended to that of capillaries. This is important because although capillary diameter changes tend to be small, capillaries represent the largest fraction of the total resistance of the retinal vascular network (capillaries cover ~6% of retina surface, compared with 2.5% by arterioles), with small changes in their diameter having proportionally large effects on blood flow rate. In one estimate from the brain, a 6% dilation in capillary diameter (~0.32 mm) generates the majority of the total blood flow increase evoked by neuronal activity, equating to a 16% increase in overall flow. In the same estimate arteriole dilations contribute just 3% to flow change.^32^ Mechanistically, activation of calcium transients within Müller cell endfeet has been associated with vasodilation of capillaries within the intermediate plexus, but not those that form the superficial or deep plexuses.^49^ This response was confirmed by manually inducing Ca^2+^ transients in Müller glia in the absence of neuronal activity and also abolished by genetically ablating of IP3R2 in the presence of light stimulus.^49^ However, light stimulus still caused vasomotor responses in the superficial plexus arterioles and capillaries, showing that Müller cell‐dependent calcium signalling only controls select capillaries within the intermediate vascular plexus.^49^ Figure 3 provides a summary of how Muller cells are thought to regulate the retinal vasculature. Muller cells receive information about neural activity because their processes contact all neural synapses. They also have processes that contact blood vessels in the deep, intermediate and superficial plexus. Vasoregulation of the intermediate plexus occurs via mechanisms involving eicosanoids such as 20‐HETE.

It is well known that Müller cells change from an early stage of diabetes. They increase expression of the intermediate filament glial fibrillary acidic protein in both human and rodent models of diabetes, indicating that gliosis of these cells occurs from an early stage of diabetes.^50^, ^51^ In addition, glutamate uptake and turnover by Müller cells is abnormal from an early stage of diabetes.^52^, ^53^ There is also functional evidence that potassium siphoning, a critical osmotic function of Müller cells, is abnormal during diabetes. Indeed, Müller cells isolated from rat retinae 4–6 months following the onset of diabetes showed reduced conductance via inwardly rectifying potassium channels (Kir4.1 channels).^54^ These functional changes are consistent with changes in expression and localization of inwardly rectifying potassium channels, with the spatial localization of Kir4.1 channels lost during diabetes.^54^ This predisposes Müller cells to swelling, and may contribute to diabetic macula oedema.

In concert with the myriad of changes observed in Müller cells during diabetes, changes in how Müller cells regulate the vasculature have also been reported.^26^, ^55^ Under normal conditions, a flickering light stimuli or activation of intracellular calcium can cause either vasodilation or vasoconstriction. However, following 4 months of diabetes, flickering light failed to induce vasodilation with the same time course and duration.^26^, ^55^ Notably, diabetes was associated with more vasoconstriction events than vasodilation, suggesting that neurovascular coupling was abnormal in diabetic retinae. In addition, calcium waves that propagated across glia (astrocytes and Müller cells) failed to induce vasodilation in diabetic retinae. While not directly attributed to Müller cells, given the later work by this group, it seems reasonable to assume that the failure of calcium dependent vascular change in the diabetic retina was at least in part due to altered Müller cells. This change in vasoregulation, may be mediated by nitric oxide (NO), a powerful regulator of neurovascular coupling.^48^ Indeed, diabetes results in increased inducible NO (iNOS) expression, and treatment that reduces iNOS expression, such as with aminoguanidine, abrogates the loss of vasodilation.

In summary, like their glia counterparts in the brain, Müller cells are important mediators of vasoregulation in response to neural activity. Diabetes is known to induce a range of changes in Müller cells from an early stage of disease that includes dysfunction of neurovascular coupling. However, it is important to note that regulation of the vasculature in response to neural needs is highly complex and Müller cells have only been associated with regulating the intermediate plexus. The regulation of the vasculature in other plexuses remains poorly understood.

## 5 NOVEL INSIGHTS INTO THE ROLE MICROGLIA IN REGULATING VASCULAR FUNCTION

The complexity of regulation of the retinal vasculature is highlighted by the observations that Müller cell processes only regulate the vessels of the intermediate plexus. The cellular contributions to regulation of the superficial and deep plexus remain poorly understood. Our recent work suggest that microglia may also regulate the retinal calibre of the retinal vasculature.^29^ Microglia are resident immune cells of the retina and central nervous system. While microglia were once thought to be largely quiescent cells that only responded to injury or disease, it is now known that microglia dynamically survey the parenchyma and play critical roles in maintaining normal neural function.^56^, ^57^, ^58^

Recent in vivo brain studies have also identified a role for microglia in regulation of cerebral blood flow.^59^, ^60^ One study showed ablation of microglia or deletion of the microglia‐specific purinergic receptor, P2yr12, impaired whisker‐stimulated neurovascular coupling in the barrel cortex.^60^ Another study similarly showed ablation of microglia or P2yr12 led to capillary dilation, increased blood flow and impaired vasodilation in the somatosensory cortex.^59^ This was recapitulated by ablation of pannexin 1 channels, which facilitate release of purines.^59^ Taken together, microglia appear to regulate neurovascular coupling in the brain via purinergic signalling, similar to ATP‐mediated neurovascular coupling by astrocytes.^46^, ^61^ Microglia may also mediate the vascular response to hypercapnia in the brain, as hypercapnia triggered microglia to produce the vasodilating agent adenosine, while hypercapnia‐induced vasodilation was abolished with loss of microglia.^60^

Apart from the most recent work, the potential contribution of microglia to vascular function has arisen from recent anatomical and functional studies that emphasise that microglia contact both capillaries and retinal synapses (Figure 2).^29^ Figure 2 shows the superficial and intermediate plexus of a mouse retina with a microglia located in close proximity. A three‐dimensional rendering of this image shows microglial processes enveloping and running along the surface of the adjacent capillary. Importantly, 73% of microglia were found to contact both blood vessels and synapses, and of the various vessel types, significantly more microglia contacted capillaries compared to larger diameter vessels. Moreover, approximately a third of microglia contacted pericyte somata, which as we have detailed above are instrumental to vasoregulation.^29^

Microglia are the only cell type in the retina that expresses the chemokine receptor, Cx3cr1, whose ligand, fractalkine, is expressed by inner retinal neurons.^62^ When fractalkine is applied to retinal explants, a prominent vasoconstriction is induced, but only in areas where microglia contacted blood vessels.^29^ Moreover, vasoconstriction is abolished when fractalkine is applied to Cx3cr1null retinal explants, or in the presence of the Cx3cr1 antagonist, AZD8797. Single cell population RNAsequencing has also been used to identify potential vasoactive substances that are released from microglia and showed that microglia express angiotensinogen, a major rate‐limiting enzyme in the formation of angiotensin II, a prominent vasoconstrictor.^29^ Moreover, application of fractalkine to retinal explants increased the expression of angiotensinogen, an effect that was abolished when applied to Cx3cr1null mouse explants. Finally, application of the angiotensin type I receptor antagonist, candesartan, prevented the change in vessel calibre induced by fractalkine. Overall, these data highlight that microglia are in the anatomical location to regulate capillaries in response to neural signals, and that fractalkine a chemokine that is expressed by neurons can induce a prominent vasoconstriction, via a mechanism involving microglial expression of the vasoactive substance angiotensinogen. A summary of the contribution of microglia to vasoregulation is shown in Figure 3, where microglia contact both neural synapses and blood vessels. They are able to induce vasoconstriction via mechanisms involving fractalkine induced angiotensin release.

## 6 MICROGLIAL‐VASCULAR INTERACTION DURING DIABETES

There is a wealth of evidence that suggests that microglia are altered during diabetes, as might be expected, for a cell type that is known to respond to disease and injury.^63^, ^64^ In human patients with moderate non‐proliferative diabetic retinopathy, microglia increase in number, change morphology and cluster in the inner retinal vasculature, particularly around areas of visible vascular pathology.^65^ Microglial activation has also been observed in animal studies showing increased microglial density and activated phenotype after 1 month of diabetes, with changes becoming increasingly more pronounced as the disease progresses.^66^, ^67^ Activated microglia are capable of releasing a range of pro‐inflammatory mediators that can promote adhesion of leucocytes and occlusion of capillaries, or neuronal degeneration. Blocking the actions of inflammatory mediators or microglia activation are known to reduce the progression of disease in rodent models of diabetes. Indeed, supressing microglial activation in animal models of diabetes, with baicalein or minocycline led to reduced production of the inflammatory mediators, IL1b and TNFa and prevented the loss of retinal ganglion cells, capillary degeneration and vascular leakage.^68^, ^69^, ^70^ Fractalkine, which is expressed by neurons, is an important regulator of microglial activation and is thought to play a role in dampening neurotoxicity in the brain and retina.^62^, ^71^, ^72^ Fractalkine expression is increased in diabetic Ins2^Akita^ mice compared to control and that subsequent genetic ablation of Cx3cr1 in mouse model of type I diabetes (i.e., Ins2^Akita^.Cx3cr1^−/−^) exacerbates neuronal loss, and promotes the release of proinflammatory mediators compared with Ins2^Akita^ wildtype mice.^72^

Beyond their role in promoting inflammation during later stages of diabetes, microglia have recently been shown to contribute to early vascular dysfunction in a diabetic rodent model.^29^ At an early stage of diabetes when there is reduced blood flow, constricted capillary diameter of the superficial vasculature and impaired vasoregulation in response to fractalkine and oxygen, microglia were found to have an increased association with capillaries. Additionally, microglia at this time (4 weeks of diabetes) were found to exhibit increased expression of angiotensinogen while retinal fractalkine levels were increased in rat retinae 4 weeks following the onset of diabetes. These data suggest that increased fractalkine early in diabetes may attenuate the renin‐angiotensin system in microglia that results in constriction of capillary vessels and inability of those vessels to respond to normal vasoregulatory stimuli. Treatment with candesartan, an antagonist to AT1 receptors, has previously been shown to reduce the development and progression of diabetic retinopathy.^73^, ^74^ The recent findings showing that anomalies in microglia‐vascular interactions occur during early diabetes are consistent with the important role of renin‐angiotensin in diabetes and may suggest the importance of a more targeted cellular treatment.^75^

## 7 CONCLUSION

In summary, diabetic retinopathy remains an important cause of irreversible vision loss in those of working age. It is now known that diabetes not only causes overt visible vascular pathology, but that it induces changes in virtually every cell type in the retina from an early stage of disease. Importantly, blood flow and vascular calibre are reduced from an early stage of diabetes, highlighting that neurovascular coupling may be abnormal. However, vascular regulation in response to neural activity is complex and likely involves at least two mechanisms (summarised in Figure 3). This is highlighted by recent studies that indicate that Müller cells regulate the calibre of blood vessels that form the intermediate plexus, whereas microglia regulate the blood flow in the superficial plexus. Moreover, abnormalities in the way Müller cells and microglia regulate vascular function in response to neural needs is likely impaired from an early stage of diabetes. These are important findings that highlight new treatment targets for managing those with diabetes. In the long term, ameliorating the early changes in vascular function that occur in those with diabetes, may be important for slowing progression. However, more detailed work is required to uncover in more detail how vascular function is regulated in response to neural activity.

## FUNDING INFORMATION

NHMRC grants APP2000669 and APP1138509 to Erica L. Fletcher, Andrew I. Jobling; ARC DP200102001 to Erica L. Fletcher.

## CONFLICT OF INTEREST

The authors declare no conflicts of interest.
